# NOVEL AUTOMATED TOOLS TO IDENTIFY COGNITIVE DECLINE AND EMOTIONAL DISTRESS IN THE CONTEXT OF MCI AND AD/ADRDS

**DOI:** 10.1093/geroni/igae098.1380

**Published:** 2024-12-31

**Authors:** Sarah Bannon, Alex Federman

**Affiliations:** Chair; Discussant

## Abstract

Alzheimer’s disease and related dementias (ADRDs) are rapidly increasing public health concerns with significant impact on individuals, families, and healthcare systems. Though recent technological advances have enabled earlier and more accurate diagnosis for some, the vast majority of individuals experience delays in diagnosis and unmet early support needs. Diagnostic delays and inadequate support exacerbate psychological distress for impacted individuals, which is important to address given 1) the impact of distress on long-term outcomes and 2) the difficulty of treating distress once it becomes chronic. Novel automated technologies have the potential to more effectively and efficiently identify changes in cognition and distress, and if leveraged could provide cost-effective avenues to improve ADRD care. This symposium describes novel methods of leveraging large-scale data and technology to enhance screening and identification of early decline and distress in the context of cognitive impairment and ADRDs. Ryan Mace, PhD will describe healthcare providers’ impressions of barriers to assessing subjective cognitive decline and gaps in current practice. Sarah Bannon, PhD will present findings from a secondary data analysis of electronic medical records examining structured data elements as indicators of psychological distress among individuals with recent ADRD diagnoses. Ayushi Divecha, MPH MPT will describe the use of random forest models to identify individuals at high risk for developing dementia from a harmonized database of 5 of the largest NIH-funded studies of aging across the US.

